# Minichromosome maintenance protein 6, a proliferation marker superior to Ki-67 and independent predictor of survival in patients with mantle cell lymphoma

**DOI:** 10.1038/sj.bjc.6602795

**Published:** 2005-09-27

**Authors:** C Schrader, D Janssen, W Klapper, J-U Siebmann, P Meusers, G Brittinger, M Kneba, M Tiemann, R Parwaresch

**Affiliations:** 1 Second Department of Internal Medicine and Hematology, University Hospital of Schleswig-Holstein, Campus Kiel, Chemnitzstr. 33, 24116 Kiel, Germany; 2 Department of Hematopathology and Lymph Node Registry, University Hospital of Schleswig-Holstein, Campus Kiel, Michaelistr. 11, 24105 Kiel, Germany; 3 Department of General Surgery, University Hospital of Schleswig-Holstein, Campus Kiel, Michaelistr. 8, 24105 Kiel, Germany; 4 Department of Medicine, Division of Hematology, University of Duisburg-Essen, Hufelandstr. 55, 45122 Essen, Germany

**Keywords:** mantle cell lymphoma (MCL), proliferation, minichromosome maintenance protein 6 (MCM6), cell cycle, Ki-67

## Abstract

Minichromosome maintenance protein 6 (MCM6) is one of six proteins of the MCM family which are involved in the initiation of DNA replication and thus represent a marker of proliferating cells. Since the level of cell proliferation is the most valuable predictor of survival in mantle cell lymphoma (MCL), we investigated lymph node biopsy specimens from 70 patients immunohistochemically with a monoclonal antibody against MCM6. The percentage of MCM6 expressing lymphoma cells ranged from 12.0 to 95.6%, with a mean of 61.0%, and was significantly higher than the percentage of Ki-67-positive cells (*P*<0.0001). Surprisingly, the ratio of MCM6-positive cells to Ki-67-positive cells was higher than in normal stimulated peripheral blood mononuclear cells, indicating a cell early G1-phase arrest in MCL. A high MCM6 expression level of more than 75% positive cells was associated with a significantly shorter overall survival time (16 months) compared to MCL with a low MCM6 expression level of less than 25% (no median reached, *P*<0.0001). Multivariate analysis revealed MCM6 to be an independent predictor of survival that is superior to the international prognostic factor and the Ki-67 index. Therefore, aside from gene expression profiling, immunohistochemical detection of MCM6 seems to be the most promising marker for predicting the outcome in MCL.

Mantle cell lymphoma (MCL) is a B-cell neoplasm characterised by a typical immunophenotype and the chromosomal translocation t(11;14) (Tsujimoto *et al*, 1984; Campo *et al*, 1999; Bertoni *et al*, 2004). The clinical course of MCL, although heterogeneous, is usually poor, with a survival time of 3–4 years. For several years a high proliferation rate, as measured by counting the number of mitotic figures or of Ki-67-expressing cells, has been recognised as a marker of poor prognosis (Lardelli *et al*, 1990; Velders *et al*, 1996; Raty *et al*, 2002). However, due to the lack of data from prospective trials and the limited predictive power of the markers available so far, cell proliferation has rarely been used for clinical decision-making in MCL. Studies of gene expression profiling demonstrated that a set of genes, designated as the proliferation signature, can predict the clinical outcome of patients with MCL with so far unrivaled precision (Martinez *et al*, 2003; Rosenwald *et al*, 2003; Ek *et al*, 2004). These studies have stimulated the search for new immunohistochemical markers of proliferation that might take the place of gene expression profiling as a predictor of outcome in MCL.

Minichromosome maintenance (MCM) proteins play an important role in the replication of eukaryotic DNA by binding to chromatin before the initiation of DNA replication (Fujita *et al*, 1997; Ogawa *et al*, 1999). Minichromosome maintenance protein 6 (MCM6) is one of six members (MCM2–7) of the MCM family (Lindner *et al*, 2002) and consists of 821 amino acids with a molecular mass of 105 kDa (Heidebrecht *et al*, 2001). We have recently developed a specific monoclonal antibody against MCM6 (Ki-MCM6) (Heidebrecht *et al*, 2001) that enables the accurate detection of MCM6 in paraffin-embedded tissue (Heidebrecht *et al*, 2001; Helfenstein *et al*, 2004). Using Ki-MCM6, Heidebrecht *et al* (2001) showed that MCM6 is detectable in nucleosols or bound to nuclear chromatin during the entire cell cycle G1, S, G2 and M phases, but is absent in G0 phase (Labib *et al*, 2001). Despite this similar expression pattern of MCM6 and Ki-67 during the cell cycle phases (positive in G1, S, G2 and M phases), detailed cell cycle analysis reveals differences between both markers. During the early G1 phase Ki-67 is undetectable, whereas MCM6 is expressed in the entire G1 phase. Therefore, a small subset of about 20% of proliferating cells in early G1 phase could be detected by MCM6 and not by Ki-67 in stimulated peripheral blood mononuclear cells (Heidebrecht *et al*, 2001).

The clinical relevance of MCM proteins as proliferation markers has been investigated immunohistochemically in several different malignant tumours (Freeman *et al*, 1999), for example, non-small-cell lung cancer (Ramnath *et al*, 2001), prostate cancer (Meng *et al*, 2001; Padmanabhan *et al*, 2004), oral squamous cell carcinoma (Kodani *et al*, 2003), chondrosarcoma (Helfenstein *et al*, 2004), oligodendroglial tumours (Wharton *et al*, 2001, 2004), oesophageal neoplasm (Going *et al*, 2002), renal cell carcinoma (Dudderidge *et al*, 2005), breast cancer (Gonzalez *et al*, 2004), endometrial carcinoma (Li *et al*, 2005) and thyroid carcinoma (Guida *et al*, 2005). Most of these studies focused on the detection of MCM2 (Freeman *et al*, 1999; Chatrath *et al*, 2003; Davidson *et al*, 2003; Kodani *et al*, 2003; Scott *et al*, 2004). So far only few investigations studied MCM6 expression (Labib *et al*, 2001; Helfenstein *et al*, 2004).

The MCM2 expression was also analysed in 36 patients with malignant B-cell lymphomas, including 11 cases with MCL (Obermann *et al*, 2005). The authors included also Ki-67 and geminin (Lygerou and Nurse, 2000; Wohlschlegel *et al*, 2000; Madine and Laskey, 2001) in their study and could demonstrate that low-grade lymphomas reside in an ‘in-cycle’ G1 state and not in G0. In this very important study of Obermann *et al* (2005), the authors did not analyse the MCM2 expression in relation to clinical data.

The aim of this study was to investigate MCM as a new proliferation marker in a large group of patients with MCL and to correlate the results with established prognostic factors for this disease. Since we have developed an antibody that accurately identifies MCM6 in paraffin-embedded tissues, an expression pattern that was tested in normal peripheral blood mononuclear cells (Heidebrecht *et al*, 2001), we chose this antibody to determine MCM protein expression in this lymphoma.

## PATIENTS AND METHODS

Biopsy specimens (lymph nodes only) from 70 previously untreated patients (Table 1) from two trials (1975 and 1985) (Brittinger *et al*, 1984; Meusers *et al*, 1989) were recut and stained with haematoxylin and eosin, Giemsa and Gomori silver impregnation. The cytology was classified according to the WHO classification of MCL into classical and blastoid subtypes (Jaffe *et al*, 2001). The diagnosis of MCL was verified by two reference pathologists from the European Mantle Cell Lymphoma Network (MT and RP).

### Antibodies and immunohistochemical staining

The immunohistochemical investigation was performed as described previously (Schrader *et al*, 2004). All sections were stained with antibodies against CD3 (dilution 1 : 20) (DAKO, Hamburg, Germany), CD5 (dilution 1 : 25) (Novocastra, Newcastle, UK), CD20 (dilution 1 : 5) (DAKO), CD23 (dilution 1 : 20) (Novocastra), cyclin-D1 (dilution 1 : 20) (Novocastra), MCM6 (dilution 1 : 1, cell culture supernatant) (Ki-MCM6, Department of Hematopathology, University of Kiel, Germany) and anti-Ki-67 (dilution 1 : 1, cell culture supernatant) (Ki-S5, Department of Hematopathology, University of Kiel, Germany). The stainings were evaluated by investigators blinded to the clinical information. In each stained section 500 neoplastic cells were counted. The MCM6 index was calculated as the percentage of positive nuclei. Only nuclear staining was counted as MCM6 positive.

Tonsil tissue was used as positive controls; negative control samples were incubated with serum instead of the primary antibody.

### Statistical methods

For the statistical tests SPSS (SPSS Inc., version 11, Chicago, IL, USA) was used. Various parameters were analysed statistically by two-sided *t*-test and the correlation coefficient, as indicated. Overall survival analysis (univariate) was analysed by the Kaplan–Meier method. Differences in significance (*P*⩽0.05) were assessed by means of the log-rank test. All clinical parameters were also compared by means of a multivariate Cox regression analysis using a stepwise (forward and backward) conditional approach.

## RESULTS

In 57 cases of MCL (81.4%) cytological analysis revealed a classical cytology and in 13 cases (18.6%) a blastoid cytological subtype. Immunophenotyping showed coexpression of CD20 and CD5 in all cases and negativity of the tumour cells for CD23. Cyclin D1 staining was positive in 69 cases (98.6%). In one case (1.4%) cyclin D1 staining could not be evaluated for technical reasons.

**Figure 1:**
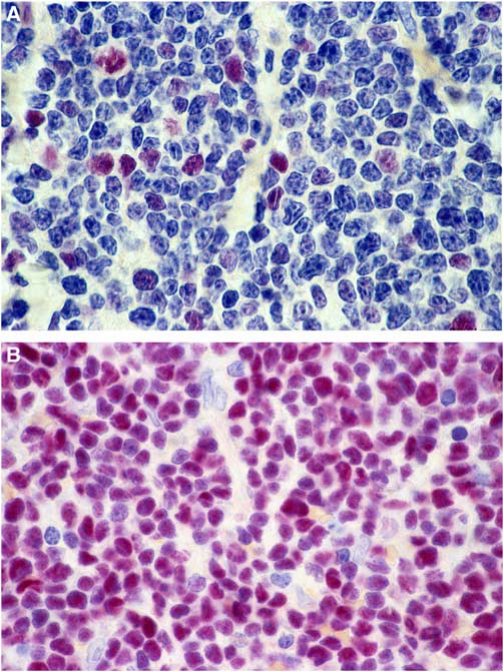
(**A**) Mantle cell lymphoma with a low level of MCM6 expression (<25%). APAAP staining, magnification × 1000. (**B**) Very high MCM6 expression (>75%). APAAP staining, magnification × 1000.

**Figure 2:**
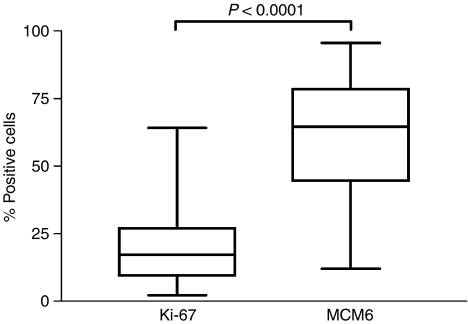
Minichromosome maintenance protein 6 and Ki-67 expression as % positive cells in all cases.

**Figure 3:**
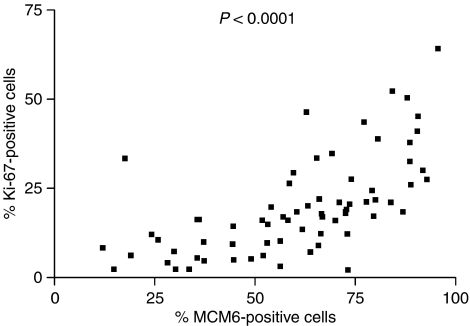
Correlation analysis of MCM6 and Ki-67 expression.

Minichromosome maintenance protein 6 staining was restricted to the cell nucleus. The expression level ranged from 12.0 to 95.6%, with a median of 64.6% and a mean of 61.0% (Figures 1A and B). We retrieved data on Ki-67 expression from our previous investigation on the same collection of samples (Schrader *et al*, 2004). As expected, the MCM6 index was significantly higher than the Ki-67 index (Figure 2, *P*<0.0001), with a mean for Ki-67 of 19.8%, compared to 61.0% for MCM6. Also not unexpectedly, the two markers showed a significant correlation (Figure 3, *P*<0.0001). Surprisingly, cytological variants like the blastoid variant did not differ in their MCM6 expression level (*P*=0.2672). In contrast, in our previous and other studies (Lardelli *et al*, 1990; Ott *et al*, 1997; Campo *et al*, 1999), the Ki-67 index was significantly higher in blastoid variants than in cases with classical cytology. By calculating the ratio of MCM6 and Ki-67, the majority of cases had high indices (mean: 4.7±4.6).

Univariate analysis of MCM6 expression in relation to clinical characteristics revealed no significant differences concerning age, sex, stage of the disease, bone marrow infiltration, extranodal involvement, lactate dehydrogenase level and IPI score (Table 1). The only significant differences concerned B-symptoms and WHO status (Table 1). A multivariate analysis of 52 patients with complete data for all variables showed that only MCM6 expression (*P*<0.0001) and IPI (*P*=0.0018) were prognostic factors for the clinical outcome (Table 2) in our group of patients.

**Figure 4:**
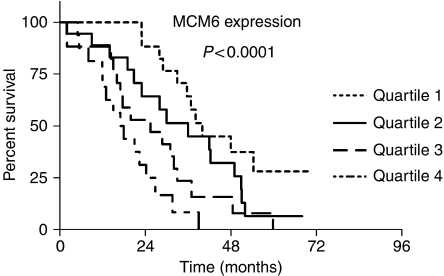
Kaplan–Meier analysis of the overall survival time of patients with MCL. Patients were ranked according to their MCM6 expression level and divided into four equal quartiles.

**Figure 5:**
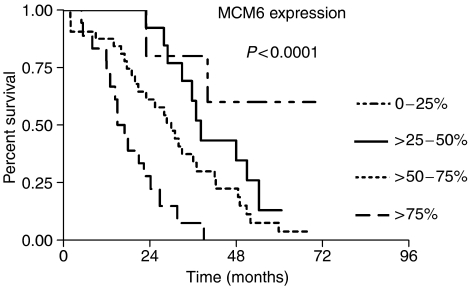
Kaplan–Meier analysis of overall survival time of patients with MCL and different MCM6 expression levels.

The patients were ranked according to their MCM6 expression level and divided into four equal quartiles (Table 3). Figure 4 shows the Kaplan–Meier analysis of overall survival time for patients in different quartiles. These data indicate that the MCM expression can identify patients with a good and a poor prognosis. The median survival times for the quartiles are listed in Table 3. In accordance with Ramnath *et al* (2001), we analysed the MCM expression in four categories. Patients with an MCM6 expression level of lower than 25% had not yet reached a median survival, in contrast to 38.2 months for the group with 25–50%, 30 months for the group with 50–75% and 16.0 months for patients with more than 75% (Figure 5 and Table 3, *P*<0.0001).

## DISCUSSION

Minichromosome maintenance proteins are components of the prereplicative complex and are essential for the initiation of DNA replication (Labib *et al*, 2001). With the development of monoclonal antibodies against MCM proteins, their use as proliferation markers for the analysis of archived tissue has become possible. To date, most studies have focused on MCM2 expression, for example, in oral squamous cell carcinomas (Kodani *et al*, 2003), prostate cancer (Meng *et al*, 2001; Padmanabhan *et al*, 2004) and ovarian serous neoplasms (Scott *et al*, 2004) chondrosarcoma (Helfenstein *et al*, 2004), oligodendroglial tumours (Wharton *et al*, 2001, 2004), oesophageal neoplasm (Going *et al*, 2002), renal cell carcinoma (Dudderidge *et al*, 2005), breast cancer (Gonzalez *et al*, 2004), endometrial carcinoma (Li *et al*, 2005) and thyroid carcinoma (Guida *et al*, 2005).

In a large study, Ramnath *et al* (2001) examined 221 patients with lung cell cancer immunohistochemically for MCM2 expression and found MCM2 expression to be an independent prognostic factor for survival. In this study, patients with MCM2 expression in less than 25% of the tumour cells had a significantly better prognosis than patients with tumours with a higher MCM2 index. Additional to the study by Ramnath, only few studies have investigated MCM proteins immunohistochemically. Brake *et al* (2003) focused on MCM7 in cervical cancer and Helfenstein *et al* (2004) on MCM6 in chondrosarcoma using the monoclonal antibody Ki-MCM6, also employed in our study.

The MCM expression in peripheral B-cell lymphomas was investigated for the first time by Obermann *et al* (2005), who could demonstrate that also in MCLs the majority of lymphoma cells reside in the cell cycle phases G1, but not in S/G2/M. These data are in line with our results of repp86 in a large series of 94 patients with MCL (Schrader *et al*, 2005). Repp86 is a spindle apparatus-associated protein expressed only during S/G2/M, absent in G1 and G0, and thus displaying the same cell cycle expression pattern like geminin (Heidebrecht *et al*, 1997, 2003; Lygerou and Nurse, 2000; Wohlschlegel *et al*, 2000; Madine and Laskey, 2001; Hodgson *et al*, 2002). We could demonstrate that the majority of the 94 cases have lower repp86 expression than Ki-67, and this indicates a G1 arrest in the subset of cases in our collective.

Mantle cell lymphomas are mature B-cell neoplasms with a poor prognosis. Measures to predict the heterogeneous clinical outcome of the disease are needed to guide clinical decision-making. Studies of gene expression profiling in MCL have stressed the importance of cell proliferation for the clinical outcome. Rosenwald *et al* (2003) summarised predictive genes for survival as the ‘proliferation signature’ since most of these genes are thought to be involved in cell cycle progression, mitosis or DNA replication. Data from gene expression profiling experiments are describing transcriptional changes of mRNA and cannot be necessarily be translated into protein expression. Furthermore, due to the high costs and technical obstacles like the need for fresh material, gene expression profiling is limited in its use for routine analysis of tumour samples. For these reasons, it is useful to confirm results based on gene expression profiling on the protein level with, for example, by immunohistochemistry. In a previous study, we studied topoisomerase II*α* expression by immunohistochemistry and were able to confirm the results from Rosenwald *et al* (Schrader *et al*, 2004). Since mRNA expression of MCM2, was reported to be associated with bad prognosis (Rosenwald *et al*, 2003), we evaluated MCM protein expression by immunohistochemistry in MCL. Out of the family of MCM proteins we chose MCM6 for our analysis, because a highly reliable monoclonal antibody against this MCM family member was available (Heidebrecht *et al*, 2001).

First we ranked the patients according to their MCM6 expression level and divided into four equal groups (quartiles). Kaplan–Meier analysis showed significant differences in the overall survival time in different quartiles (Figure 4, Table 3, *P*<0.0001). Second, for the Kaplan–Meier analysis, we chose different MCM6 expression levels according to the categories of Ramnath *et al* (2001). In the group under 25% MCM6-positive cells, the median overall survival time was despite the long follow-up time not reached, indicating that this group of patients has an excellent prognosis. The other patients with higher MCM6 expression had a significantly shorter overall survival, with a median of 38.2 months in the group >25–50% positive cells compared to the group representing >50–75% MCM6-positive cells, which had a median of 30.0 months. The highly proliferating group of 75% and more MCM6-positive cells had a median survival time of only 16 months (*P*<0.0001). In the multivariate Cox regression analysis which included Ki-67, only MCM6 expression (*P*<0.0001) and IPI score (*P*=0.0018) proved to be prognostic factors for clinical outcome of patients with MCL. These results clearly indicate that MCM6 expression is a much stronger predictor of overall survival in MCL than Ki-67. Further, the data confirm the data from gene expression profiling studies that MCM protein expression can predict overall survival in MCL. It will be interesting to evaluate if MCM2 expression by immunohistochemistry is as effective as MCM6 expression reported here.

Minichromosome maintenance protein 6 expression in MCL ranged from 12.0 to 95.6%, with a mean of 60.9%. In contrast, the Ki-67 index was significantly lower, with a mean of 19.8% and a range between 2.2 and 64.2%. Nevertheless, the Ki-67 and MCM6 indices showed a good correlation (Figure 3, *P*<0.0001). These data are in line with others (Ramnath *et al*, 2001; Helfenstein *et al*, 2004), indicating that the ratio of MCM6- and Ki-67-positive cells is larger in MCL than the Ki-67 expressing cells. Blastoid variants usually have a more aggressive clinical behaviour than classical types, and the proliferation indices in the blastoid subtypes are usually higher (Schrader *et al*, 2004). To our surprise, there were no significant differences in MCM6 expression (*P*=0.27) between the different cytological subtypes. Although both markers MCM6 and Ki-67 are expressed in the G1, S, G2 and M phases of the cell cycle, the higher number of MCM6-positive cells can be explained by the expression of MCM6 in the early G1 phase, when Ki-67 expression cannot be detected (Heidebrecht *et al*, 2001). Compared to stimulated peripheral blood mononuclear cells (ratio: mean: 1.4±0.5; Heidebrecht *et al*, 2001), the difference between Ki-67 and MCM6 positive cells in our series of MCLs is higher (mean: 4.7±4.6). These data show that a subset of mantle cell tumour cells is arrested in the early G1 phase and the MCM6-positive cells do not necessarily represent the real proliferating compartment of the lymphoma. This fact could also explain why the blastoid and classical subtype had no significant difference in the MCM6 expression as a marker for the G1 arrest and not as a proliferation marker. As this cell cycle arrest has been shown by others (Stoeber *et al*, 2001; Eward *et al*, 2004; Obermann *et al*, 2005; Tachibana *et al*, 2005), we could demonstrate that this has a clinical relevance in patients with MCLs. Our data on expression of repp86 in MCL (Schrader *et al*, 2005) showed that real proliferation activity is an important prognostic factor in MCL. On the other hand, patients with cell arrest (G1 arrest) indicated by high MCM6 expression also predict survival in this disease.

The typical translocation t(11;14)(q13;q32) of MCLs causes an overexpression of the cell cycle regulating protein cyclin D1, that binds to the cyclin-depending kinases (CDK) 4 and 6 and therefore plays an important role in the control of the G1 phase in the cell cycle (Hunter and Pines, 1994). The cyclin/CDK complexes are important for progression of cells into S phase. Taking this molecular mechanism in consideration, it is more surprising that a majority of MCL tumour cells are not in S–M–G2 phases, but in G1 arrest. Future research has to combine markers of cell proliferation and cell cycle arrest with markers of other biological processes like apoptosis (Martinez *et al*, 2003) to get insight into the biology and explain the heterogeneous therapeutic response of this disease.

In conclusion, high MCM6 expression indicates early G1-phase arrest, and is a new prognostic marker in MCL superior to other clinical prognostic parameters and Ki-67 expression.

## Figures and Tables

**Table 1:** Univariate analysis of prognostic factors in relation to MCM6 expression

		**Overall survival**		**MCM6 expression (%)**			
	***N***	**Median (months)**	***P*-value**	**Mean±s.d.**	**Median**	**Range**	***P*-value**
*Age*							
<60	28	37	0.002	55.5±23.5	55.0	14.8–90.6	0.098
>60	42	23		64.6±19.8	66.2	12.0–95.6	

*Sex*							
Male	57	29	0.688	61.0±21.3	65.4	12.0–95.6	0.605
Female	13	28.8		58.1±24.2	60.4	14.8–90.4	

*B-symptoms*							
Yes	25	17.0	0.002	67.8±18.5	70.0	25.8–91.8	0.027
No	43	35.7		55.9±22.3	57.0	12.0–95.6	

*BM infiltration*							
Yes	42	28.0	0.138	62.1±19.7	65.6	12.0–90.6	0.488
No	27	33.0		58.2±24.6	57.0	14.8–95.6	

*Stage*							
1+2	5	42.3	0.249	57.3±19.6	56.3	33.6–87.9	0.638
3+4	59	28.0		62.1±21.9	66.0	12.0–95.6	

*Status (WHO)*							
0–1	53	31.0	0.013	58.3±21.7	60.4	12.0–95.6	0.014
⩾2	15	19.9		73.5±16.6	74.0	35.6–90.6	

*Extranodal*							
Yes	58	29.0	0.157	60.6±21.5	65.7	12.0–92.8	0.773
No	12	28.8		62.6±23.7	62.3	14.8–95.6	

*LDH*							
<240	51	29.0	0.043	60.6±21.5	63.2	12.0–95.6	0.055
>240	13	17.9		73.1±15.4	73.2	37.2–91.8	

*IPI*							
0–1	17	40.0	0.007	53.0±22.9	56.3	14.8–87.9	0.082
⩾2	53	25.4		63.5±20.9	66.4	12.0–95.6	

BM=bone marrow; stage=Ann Arbor stage; status=performance status; extranodal=extranodal involvement; LDH=lactate dehydrogenase; IPI=International Prognostic Index; full data are not availble for all patients.

**Table 2:** Uni- and multivariate Cox regression analysis of all prognostic factors with respect to overall survival in 52 MCL patients with complete data available (out of 70 patients)

		***P*-value**	
**Characteristics**	**Reference level**	**Univariate**	**Multivariate**
MCM6 expression	0–25% *vs* >25–50% *vs* >50–75% *vs* >75%	<0.0001	<0.0001
Ki-67 expression	⩽10% *vs* >10%	0.0045	0.2473
International prognostic index	0–1 *vs* ⩾2	0.0011	0.0018
LDH	Normal *vs* elevated	0.0841	0.7638
Age	<60 *vs* >60	0.0083	0.5001
Sex	Male *vs* female	0.6679	0.8449
B-symptoms	Yes *vs* no	0.0241	0.4884
Bone marrow infiltration	Yes *vs* no	0.0650	0.9970
Stage	1+2 *vs* 3+4	0.6677	0.7943
Performance status (WHO)	0+1 *vs* ⩾2	0.0246	0.4430
Extranodal involvement	Yes *vs* no	0.0749	0.3797

**Table 3:** Results of the analysis of MCM6 expression in relation to the overall survival time in 70 patients with MCL

**MCM6 expression**	***n* (%)**	**Median OS (months)**	**5-year survival (%)**	***P*-value**
Quartile 1 12.0–44.7%	18 (25.7)	40.0	22.2	<0.0001
Quartile 2 49.0–65.4%	18 (25.7)	36.0	11.1	
Quartile 3 65.8–77.8%	17 (24.3)	25.4	0	
Quartile 4 79.2–95.6%	17 (24.3)	17.5	0	

0–25%	5 (7.1)	Not reached	60.0	<0.0001
>25–50%	14 (20.0)	38.2	14.3	
>50–75%	32 (45.7)	30.0	6.3	
>75–100%	19 (27.1)	16.0	0	

OS=overall survival time.
